# How the Oviduct Lipidomic Profile Changes over Time after the Start of an Obesogenic Diet in an Outbred Mouse Model

**DOI:** 10.3390/biology12071016

**Published:** 2023-07-17

**Authors:** Kerlijne Moorkens, Jo L. M. R. Leroy, Jusal Quanico, Geert Baggerman, Waleed F. A. Marei

**Affiliations:** 1 Gamete Research Centre, Laboratory for Veterinary Physiology and Biochemistry, Department of Veterinary Sciences, University of Antwerp, 2610 Wilrijk, Belgium; jo.leroy@uantwerpen.be (J.L.M.R.L.); waleed.marei@uantwerpen.be (W.F.A.M.); 2 Centre for Proteomics, University of Antwerp, Groenenborgerlaan 171, 2020 Antwerp, Belgium; jusal.quanico@uantwerpen.be (J.Q.); geert.baggerman@vito.be (G.B.); 3 Health Unit, Flemish Institute for Technological Research (VITO), Boeretang 200, 2400 Mol, Belgium; 4 Department of Theriogenology, Faculty of Veterinary Medicine, Cairo University, Giza 12211, Egypt

**Keywords:** obesity, infertility, high-fat/high-sugar diet, oviductal epithelium, lipidomics, MALDI-MSI

## Abstract

**Simple Summary:**

This study investigated the lipidomic changes in the oviduct at different time points (from 3 days to 16 weeks) during feeding an obesogenic diet, in an outbred mouse model. We used MALDI mass spectrometry imaging focusing on changes in the oviductal epithelium (OE). The obesogenic diet resulted in an overall higher average peak intensity of all detected lipids, and we could identify differentially regulated lipids (DRLs) already after 3 days. The number of DRLs progressively increased and became more persistent after long-term obesogenic diet feeding. Functional annotation revealed that the alterations were mainly in phospholipids, sphingomyelins and lysophospholipids.

**Abstract:**

We investigated whether a high-fat/high-sugar (HF/HS) diet alters the lipidomic profile of the oviductal epithelium (OE) and studied the patterns of these changes over time. Female outbred Swiss mice were fed either a control (10% fat) or HF/HS (60% fat, 20% fructose) diet. Mice (*n* = 3 per treatment per time point) were sacrificed and oviducts were collected at 3 days and 1, 4, 8, 12 and 16 weeks on the diet. Lipids in the OE were imaged using matrix-assisted laser desorption ionisation mass spectrometry imaging. Discriminative *m*/*z* values and differentially regulated lipids were determined in the HF/HS versus control OEs at each time point. Feeding the obesogenic diet resulted in acute changes in the lipid profile in the OE already after 3 days, and thus even before the development of an obese phenotype. The changes in the lipid profile of the OE progressively increased and became more persistent after long-term HF/HS diet feeding. Functional annotation revealed a differential abundance of phospholipids, sphingomyelins and lysophospholipids in particular. These alterations appear to be not only caused by the direct accumulation of the excess circulating dietary fat but also a reduction in the de novo synthesis of several lipid classes, due to oxidative stress and endoplasmic reticulum dysfunction. The described diet-induced lipidomic changes suggest alterations in the OE functions and the oviductal microenvironment which may impact crucial reproductive events that take place in the oviduct, such as fertilization and early embryo development.

## 1. Introduction

The prevalence of obesity among women of reproductive age has been significantly increasing worldwide, mainly due to the overconsumption of obesogenic (high-fat/high-sugar, HF/HS) diets and a sedentary lifestyle. This is strongly linked with reduced fertility [1]. Overweight and obese women are more likely to have lower fertilization rates, poor oocyte and embryo quality, and higher rates of miscarriage compared to normal-weight women [2,3]. Several studies have demonstrated that most pregnancy losses occur very early, in the first 2 weeks after fertilization, suggesting early preimplantation embryo mortality and failure of implantation [4,5]. This is usually attributed to reduced oocyte quality [3,6,7]. However, alterations in the oviductal microenvironment may also impact early embryo development and increase the risk of early embryonic losses [8]. Several crucial reproductive events take place in the oviduct, such as oocyte and sperm capacitation, fertilization, early embryo development, embryo genome activation and epigenetic (re)programming [8,9,10,11]. Nevertheless, very little is known about the potential effects of an obesogenic diet and obesity on the oviduct and its microenvironment.

High-fat/high-sugar (HF/HS)-diet-induced obese mouse models are frequently used to study the pathogenesis of obesity and its complications [12]. The consumption of obesogenic diets has been shown to induce acute responses, as early as after three days of feeding, where mice exhibit reduced glucose tolerance, and increased blood cholesterol and inflammatory cytokines [13,14]. Prolonged feeding of an obesogenic diet leads to insulin resistance and increases hyperlipidaemia, which increases adiposity and accumulation of lipids in non-adipose tissues, causing lipotoxicity and oxidative stress which eventually results in cellular dysfunctions [15,16,17,18,19]. More specifically, diet-induced changes in the lipid content of different tissues such as the liver, heart and muscles have been described, which is associated with alterations in cellular metabolism and functions and an increased risk of various metabolic disorders [20,21,22,23]. These tissues are metabolically highly active and might be more sensitive to changes in circulating lipids. Whether the same changes also occur in the reproductive tissues is not known. Some studies have shown evidence of increased lipid content in the ovarian follicular fluid microenvironment and the oocytes in diet-induced obese mouse models [24,25,26,27,28,29,30,31,32] and in women [33,34,35]. Many of these studies have demonstrated a direct lipotoxic impact on oocyte developmental competence. Furthermore, the effect on ovarian functions appears to be mainly induced by the obesogenic diet per se regardless of the development of the obese phenotype [36]. On the other hand, the potential effects of HF/HS-diet-induced obesity on the oviductal microenvironment and its lipid content have not been elucidated in humans or in relevant animal models.

The sensitivity of the oviduct to hyperlipidemia has been examined only in a few studies. Free fatty acid (FFA) concentrations in the blood were shown to be correlated to those in the oviductal fluid in cattle [37] and exposure of the oviductal cells in vitro to high FFA concentrations altered the oviductal cell proliferation and integrity, and sperm binding capacity [38]. Data from studies in which bovine embryos, in vitro produced under standard conditions, were transferred to the oviducts of cows suffering from high blood FFA concentrations due to negative energy balance, indicate that the oviducts of these cows may be compromised in their ability to support early embryo development, which may contribute to lower survival rates of the embryos compared to the embryos transferred to healthy cows [39,40]. In addition, we have recently shown that feeding an HF/HS diet in mice upregulated genes involved in endoplasmic reticulum stress responses and in oxidative stress in the oviductal epithelial cells only after 3 days of feeding [41]. These responses reoccurred and were aggravated during later phases of HF/HS feeding and were associated with marks of inflammation after long-term feeding and the development of obesity [41]. Whether such impact is linked to alterations in the lipid profile of the oviduct is not known yet. 

Importantly, feeding an obesogenic diet is not only expected to increase neutral lipid accumulation in somatic cells, but may also change the composition of lipids that play very important roles in the structure and functions of the plasma membrane, membranous organelles, and in cell signalling and cell-to-cell interaction [42]. Alterations in the dietary fat content and in the ratio between saturated and unsaturated fatty acids could influence the lipid profile of the ovarian follicular fluid, follicular cells and endometrial epithelial cells in cows and sheep [43,44,45,46]. This has not been illustrated in the oviduct while it is now generally accepted that biochemical alterations in the oviduct can be critical for early embryo development [47,48]. The oviductal cells and fluid are rich in lipids, such as cholesterol; high- (HDLs) and low-density lipoproteins (LDLs), triglycerides and fatty acids [49], as well as phospholipids and sphingolipids [50,51], which can significantly influence the oviductal and embryonic cell signalling and molecular functions [52]. 

Taken together, a profound understanding of how diet and obesity can influence the oviductal lipidomic profile is important for increasing awareness and implementing preventative measures during the periconceptional period to enhance fertility. Advances in the matrix-assisted laser desorption ionization mass spectrometry imaging (MALDI MSI) technique have enabled the analysis of the spatial distribution of small molecules such as lipids in tissues of interest [53], and the technique was successfully used to describe the spatial distribution of different lipid classes in the ovarian follicles [54]. The aim of this study was to investigate whether an HF/HS diet can alter the lipidomic profile of the oviductal epithelium and to study the patterns of these changes in the oviductal epithelial layer over time as the mice continue to consume such an obesogenic diet and develop an obese phenotype. This was examined in an outbred mouse model. 

## 2. Material and Methods

### 2.1. Ethical Approval

All procedures in this study were approved by the ethical committee of the University of Antwerp and performed accordingly (ECD approval number no. 2014-57). All methods were performed in accordance with the relevant ethical guidelines and regulations. This study complies with the ARRIVE guidelines [55]. 

### 2.2. Experimental Animals, Diet and Experimental Design

Five-week-old, non-pregnant, sexually mature female outbred Rj:Orl Swiss mice (hereafter referred to as “Swiss” mice, *n* = 42, Janvier labs) were used. The mice were randomly divided into two groups with ad libitum access to either a control diet (CTRL) or a high-fat/high-sugar diet (HF/HS). An HF/HS diet is more representative of the typical Western-style diet, compared to a high-fat diet (HFD) [56]. The HF/HS group was fed with a 60 kJ% fat (beef tallow) and 9.4% sucrose diet (E15741-34, Sniff diets, Soest, Germany) in combination with drinking water containing 20% fructose (Merck, 102109450). Beef tallow was chosen since it contains more saturated fat compared to lard. Fructose was chosen over glucose and sucrose because high consumption of fructose is known to cause insulin resistance and obesity in rodents [57]. A high-fat/high-fructose diet can thus induce metabolic syndrome and type 2 diabetes in mice [56]. Furthermore, it has been shown that fructose has a reduced capacity to stimulate satiety and it also appears to be a better inducer of metabolic syndrome compared to glucose [58]. 

Mice in the control group were exposed to a matched, purified (not a grain-based chow diet) CTRL diet (E157453-04, Sniff diets, Soest, Germany), containing 10 kJ% fat and 7% sucrose, and had ad libitum access to fructose-free water. This CTRL diet is also lower in saturated fatty acids. Experimental feeding continued for up to 16 weeks. The food intake of all mice was monitored and the mice were weighed weekly during the whole experiment. 

Mice were euthanized by decapitation at 6 different time points (*n* = 3 per treatment per time point): 3 days, 1 week (1 w), 4 w, 8 w, 12 w and 16 w after the start of feeding the CTRL or the HF/HS diet. Oviducts were collected as explained below to study different outcome parameters. Female mice were exposed to bedding from male cages 24 h before euthanasia to synchronize their oestrous cycles (Whitten effect) [59] and to collect the samples during the follicular phase (before ovulation), ensuring no follicular cells or oocytes were present in the oviduct at sample collection. The time points at which samples were collected are based on the results of previous studies where multiphasic acute and long-term effects of an HFD on general metabolic features in mice have been reported [13]. 

### 2.3. Assessment of Live Body Weight

The weight of each mouse was recorded weekly. The weight gain data are derived from 21 mice per dietary group at the first time point. The number of mice was reduced after each time point due to the culling of a subset of animals (*n* = 3) at each time point for sample collection.

### 2.4. Oviduct Collection

At each time point, mice were dissected and the oviducts were collected in L15 medium (Thermo Fisher Scientific, Belgium) supplemented with 50 IU/mL penicillin G sodium salt (Merck, Belgium). Fat and surrounding tissue were removed under a stereomicroscope. The oviducts were straightened by dissecting the surrounding connective tissue and ligaments, and trimmed from both sides to remove the infundibulum and isthmus regions [60]. From each mouse, one oviduct was used for lipidomic analysis, while the other oviduct was used to collect oviductal epithelial cells (OECs) for gene expression analysis, the data of which is already published in Moorkens et al. (2022) [41]. The ampullary parts of oviducts that were used for lipidomic analysis were then washed two times in phosphate-buffered saline (PBS; Life Technologies, Belgium), wrapped straight in a piece of aluminium foil, snap frozen in liquid nitrogen and stored at −80 °C. 

### 2.5. Assessment of the Lipid Distribution in the OE Using MALDI Mass Spectrometry Imaging (MALDI MSI)

Matrix-assisted laser desorption ionisation (MALDI) imaging was used for the imaging of lipids that are present in the mouse oviductal tissue. 

### 2.6. Reagents

Carboxymethylcellulose (CMC), norharmane, red phosphorus, ammonium acetate (NH_4_OAc 7.5 M solution) and formic acid (FA) were obtained from Sigma (Merck Life Science B.V., Overijse, Belgium, molecular grade). LC-MS-grade acetone, isopropanol (IPA), methanol (MeOH) and water were obtained from Biosolve B.V. (Valkenswaard, The Netherlands), while *n*-hexane was obtained from VWR International BVBA (Leuven, Belgium). 

### 2.7. Sample Preparation and Processing and Imaging Data Acquisition

The straightened oviduct segments were first embedded in 2% carboxymethyl cellulose (CMC) after which they were stored at −80 °C. The embedded oviduct segments were then thawed from −80 °C to −20 °C for 30 min, after which 10 μm oviductal ampullary sections were obtained using a cryostat (Leica Microsystems, Belgium BVBA). The sections were mounted on indium tin oxide (ITO)-coated slides (LaserBio Labs, Sophia-Antipolis, Valbonne France) and dried under vacuum in a desiccator for 15 min. Norharmane was used as a matrix as it is suitable for both negative and positive reflectron modes [61]. Sublimation of the slide with matrix was necessary for the successful generation of ions by the absorption of laser irradiation. The matrix was sublimated for 13 min using a sublimation setup assembled in-house, composed of a sublimation apparatus (Merck) that was heated at 140 °C with an oil bath (Filter Service NV, Eupen, Belgium).

Lipid imaging was performed using a RapifleX MALDI Tissue-typer TOF mass spectrometer (Bruker Daltonics, Bremen, Germany). The instrument was equipped with a Smartbeam 3D Nd:YAG (355 mm) laser capable of firing up to 10 kHz and was controlled using FlexControl 4.0 (Build 46) software (Bruker Daltonics). The single smartbeam parameter was set to a 6-µm × 6-µm scan range with a resulting field size of 10 µm. Spectra at the scan range of *m*/*z* 400–2000 were obtained in positive and negative reflectron mode at a laser repetition rate of 10 kHz. Both acquisition modes were used to detect all possible lipids that ionise better in either the positive or the negative reflectron mode. The sampling rate was set to 1.25 GS/s. The laser power was adjusted using test shots at random positions on the sample in order to reach the optimal ionization threshold. A total of 500 laser shots were accumulated for each raster point. The deflection of masses below *m*/*z* 400 was activated by default. Spectra were acquired at a lateral resolution of 10 μm. External calibration was performed using the PepMix 6 standard (LaserBio Labs) and red phosphorus adducts in positive and negative modes, respectively. 

### 2.8. Assessment of the Different Histological Regions in the Oviductal Cross-Section Using Histological Staining

A bright field image was obtained for each section used for MALDI MSI after hematoxylin and eosin (H&E) staining. For that, the matrix was first removed by submerging the slide in 70% then 100% isopropanol (Carl-Roth, Karlsruhe, Germany). The slides were placed in an acetic acid formaldehyde (VWR, Leuven, Belgium) solution for 2 min (min), after which they were dipped five times in tap water and then AquaDest. H&E staining was then performed following standard procedures [62]. Finally, the sections were covered with DPX mount medium and a cover slip and stored at room temperature. Images were made of the H&E-stained sections and these images were used to align (co-register) the MALDI MSI images to identify the epithelial cell layer of the oviduct as described below. 

### 2.9. Lipidomic Data Acquisition and Analysis of the Oviductal Epithelial Cell Layer

The MSI data sets were analysed with SCiLS Lab 3D, version 2016b (Bruker Daltonics) [63]. SCiLS reduces the impact of systematic and technical variations to improve the reproducibility of MALDI MSI experiments. The baseline was corrected using the Tophat morphological algorithm and the data were normalized based on the total ion count (TIC) method [64]. Orthogonal matching pursuit (OMP) was used for peak detection and the peaks were aligned to the mean spectrum by centroid matching [65]. 

A first overview of the spatial regions of the luminal epithelial cells and stroma in the cross sections of the oviduct along the ampulla was achieved through spatial segmentation by applying the bisecting k-means algorithm using the Manhattan distance metric. The depth of clustering was selected interactively. The cluster corresponding to the epithelial cell regions was identified by co-registering the MS images with the optical scans of the same section after it had been subjected to H&E staining. Spectra from this cluster were subjected to receiver operating characteristic (ROC) analysis at each time point to detect *m*/*z* values that were discriminative between the HF/HS and CTRL groups. The ROC curve, which plots sensitivity versus 1-specificity [66] is a commonly used measure to assess how well a test or model can discriminate individuals, such as the detected masses, into two classes [67], such as HF/HS and CTRL in the case of our study. To avoid the technical bias that may occur towards the detection of more abundant lipid masses, the ROC analysis was performed in two directions: HF/HS versus CTRL and CTRL versus HF/HS. The analysis was also followed by MS/MS and database search to identify the detected differentially regulated lipids (DRLs) as described by Bertevello et al. (2018) [68].

### 2.10. Statistical Analysis

Each individual mouse is considered an experimental unit. Statistical analysis of live body weight was carried out using IBM Statistics SPSS (IBM SPSS statistics version 26). The numerical data were checked for homogeneity of variance using Levene’s test. Live body weight was analysed using repeated measures ANOVA to study the main effects and interaction of treatment and time. In addition, two-tailed independent sample t-tests were performed to study the effect of treatment on body weight at each time point. As previously mentioned, MSI data sets were analysed with SCiLS Lab 3D, version 2016b (Bruker Daltonics).

The receiver operating characteristic (ROC) analysis was used to evaluate whether a detected lipid signal was discriminative between the CTRL and HF/HS OE. For this, two directions of change were tested: HF/HS vs. CTRL and CTRL vs. HF/HS. A discrimination threshold of 0.7 was used for the area under the curve (AUC) of the ROC plot, above which a mass is considered discriminative or differentially regulated between the two treatment groups. A representative ROC plot can be found in the Supplementary Materials (Figure S1). Masses with an AUC ≥ 0.7 in the HF/HS vs. CTRL analysis were higher in abundance in the HF/HS group compared to the CTRLs, and vice versa.

## 3. Results

### 3.1. The Impact of the HF/HS Diet on Live Body Weight

**Figure 1:**
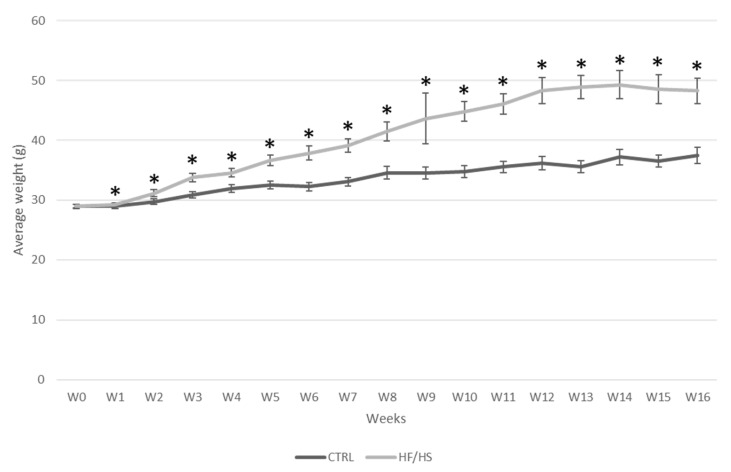
The effect of HF/HS diet on body weight in Swiss mice. Experimental feeding was started at 5 weeks of age (W0) for 21 mice per dietary group at the first time point. Body weight was monitored weekly. The number of mice was reduced after each time point due to the culling of a subset of animals (*n* = 3) at each time point for sample collection. Data are shown as mean ± SEM. Significant differences (*p* ≤ 0.05) between treatment groups per time point are indicated by an asterisk (*).

A significant treatment x time effect of the HF/HS diet consumption on body weight was shown (*p* < 0.001) using repeated measures ANOVA. The difference in body weight between the HF/HS and the control group was already significant from 1 w onwards. The increase in body weight in the HF/HS group reached a plateau at 12 w of feeding where the mice were about 33.6% heavier than those in the control group (48.30 g ± 2.14 vs. 36.16 g ± 1.17) (Figure 1).

### 3.2. Distinct Lipid Distribution in the OE

**Figure 2:**
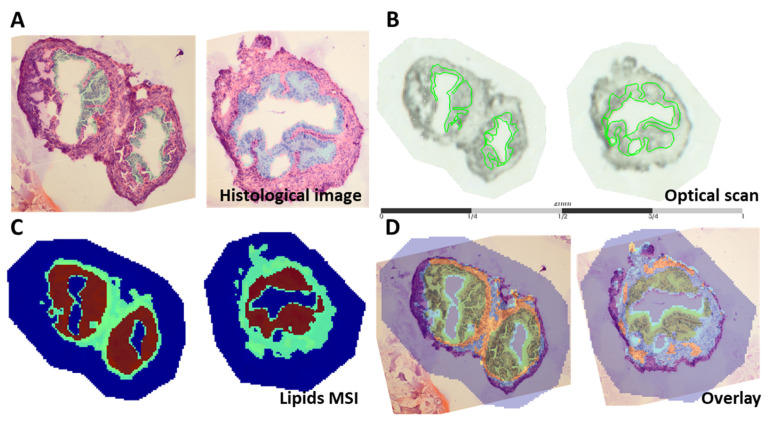
MALDI MSI analysis of two representative oviductal cross-sections at the 16-week time point. (**A**) Histological images of the oviductal cross-sections with the region of interest (ROI; the epithelium) highlighted in blue and (**B**) the co-registration of the MS images with the optical scans with the ROI indicated in green. (**C**) Spatial segmentation maps of the whole acquired spectrum with each ROI arbitrarily assigned a particular colour to show specific clusters associated with the OE (highlighted in brown) and oviductal stroma (highlighted in green). (**D**) Histological image overlaid (overlay) on spatial segmentation maps.

The OE was highlighted as the region of interest on H&E staining images (Figure 2A) as well as on the optical scans (Figure 2B). Spatial segmentation, a method that generates a segmentation map by grouping spectra acquired in an imaging experiment by similarity using a clustering algorithm (bisecting k-means), was applied to the imaging datasets (Figure 2C). This method showed that spectra acquired from regions associated with the oviductal epithelia (brown) are distinct from those acquired from regions associated with the stromal (green) regions, and can be isolated for further statistical analyses. An overlay of the H&E image and the spatial segmentation maps was used for alignment and to focus the subsequent analysis on the epithelial layer (Figure 2D). 

**Figure 3:**
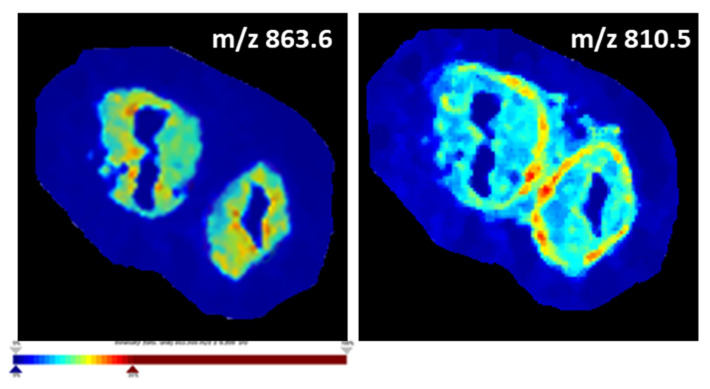
Ion density maps plotting the distribution of specific lipid masses, *m*/*z* 863.6 and *m*/*z* 810.5, based on their intensity across the entire tissue section showing their preferential localization to the OE (*m*/*z* 863.6) and the oviductal stroma (*m*/*z* 810.5). The intensity levels of the *m*/*z* values are presented by a colour gradient going from dark blue, corresponding to the lowest intensity (i.e., low abundance of that specific lipid mass in a particular spot), to dark red, showing the highest intensity.

Ion density maps indeed demonstrate the preferential localization and variable abundance of specific lipid species in either the OE or oviductal stroma. For example, Figure 3 demonstrates that *m*/*z* 863.6 is more abundant in the mucosa (represented by an orange-red coloured OE) compared to the oviductal stroma (which is green-blue coloured), whereas *m*/*z* 810.5 is more abundant in other parts of the stroma (represented by orange-red colour in this region) and less abundant in the oviductal mucosa (displayed by the light blue colour).

### 3.3. The Effect of the HF/HS Diet on the Average MALDI MSI Peak Intensity at Different Time Points

**Figure 4:**
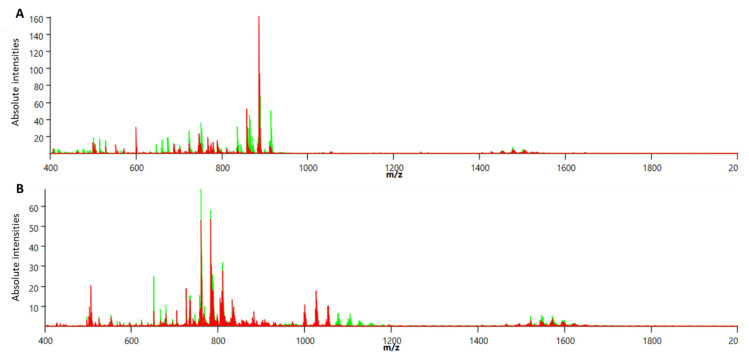
Representative skyline projection spectra of MALDI MSI analysis of the oviductal epithelium sections in negative (**A**) and positive (**B**) reflectron mode. CTRL samples are represented by green peaks; HF/HS samples are represented by red peaks. *Y*-axis shows the absolute intensity of the detected peaks or *m*/*z* (mass-to-charge ratio) signals that are shown on the *X*-axis.

MALDI imaging analysis of the oviductal sections was performed in both positive and negative reflectron modes. Skyline projection spectra were generated from all detected ion signals in the scan range of *m*/*z* 400–2000 coming from all CTRL or all HF/HS oviductal epithelium sections at each time point. The skyline projection spectra show numerous peaks with variable intensity in both negative (Figure 4A) and positive (Figure 4B) modes. The *m*/*z* peak coming from matrix clusters is represented in both spectra at the *m*/*z* interval of *m*/*z* 500 and was excluded (interval size of 14 *m*/*z*) from the analysis. 

**Figure 5:**
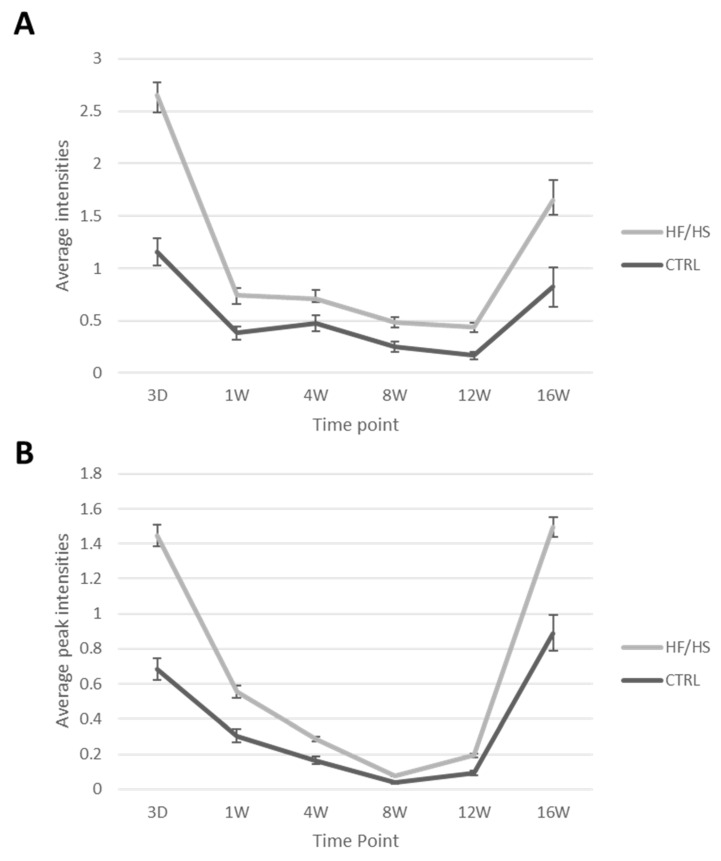
The average peak intensity of all signals coming from CTRL and HF/HS oviductal epithelia over the different time points in negative (**A**) and positive (**B**) reflectron mode. Data are shown as mean ± SEM.

The overall average peak intensity of all detected signals on the spectra varied from one time point to another. This could be due to technical reasons such as matrix deposition variation and detector sensitivity variation which are difficult to avoid in lipidomic analysis. All control and HF/HS samples of each time point were always processed, imaged and analysed together in one batch. Therefore, we focus on the relative comparison between the control and the HF/HS samples at each time point. The results show that the HF/HS OE exhibit a higher overall average peak intensity at all time points compared to the CTRL OE (Figure 5). At 3 days of feeding, the average peak intensity of the HF/HS OE cells was more than twofold higher than that of the CTRLs. In both acquisition modes, this difference was less pronounced from 1 to 12 weeks and then increased again at 16 weeks. 

## 4. DRLs Induced by the HF/HS Diet at Each Time Point

### 4.1. ROC Analysis of Spectra

Receiver operating characteristic (ROC) analysis on the spectra of the OE revealed a progressive change in the lipid profile of the OE in response to HF/HS diet feeding over time, compared to the CTRLs. This was shown by an increase in the total number of detected discriminative masses (DMs) over the different time points for both acquisition modes and in both directions of the ROC analysis (HF/HS vs. CTRL and CTRL vs. HF/HS) (Table 1). A total of 11 masses were discriminative already after 3 days of feeding. This increased to 74 masses at 1 week and stayed in this range until week 12. A marked increase in the number of DMs (227) was then detected at 16 w of feeding. The majority of these DMs were more abundant in the CTRL OE compared to the HF/HS OE (i.e., were detected in the CTRL vs. HF/HS comparison).

**Figure 6:**
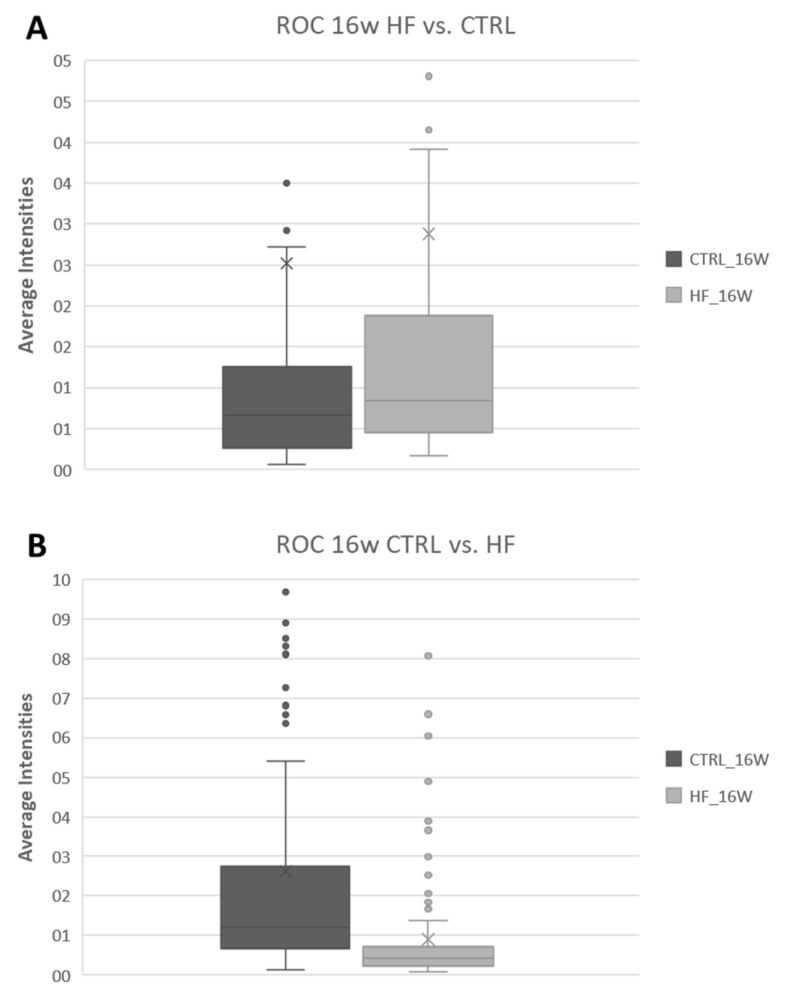
Representative boxplots showing the distribution of the average intensity of the peaks of ROC-detected DRL in the OE in either the HF/HS vs. CTRL (**A**) or CTRL vs. HF/HS (**B**) comparison after 16 w of feeding. (Legend: HF = HF/HS.)

In order to illustrate and confirm this difference, the overall average intensity of all DMs detected at 16 w (as a representative example) in the HF/HS vs. CTRL comparison (45 DMs) and in the CTRL vs. HF/HS comparison (182 DMs) are plotted in Figure 6 to show the differences in their intensities between the HF/HS and CTRL groups. HF/HS vs. CTRL DMs indeed exhibit higher intensities in the HF/HS group, and vice versa.

**Figure 7:**
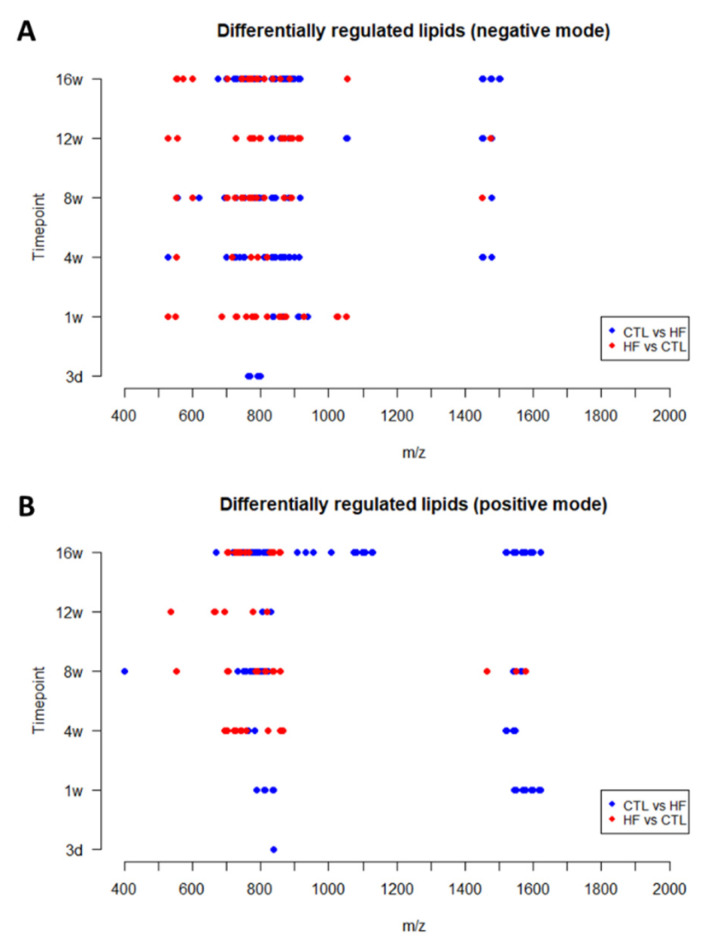
Distribution of molecular weight (*m*/*z*) of the DRLs across all time points detected in negative (**A**) and positive (**B**) reflectron mode. (Legend: HF = HF/HS; CTL = CTRL.)

In both acquisition modes, the detected DRLs across all time points were focused in specific mass ranges (Figure 7). Most peaks were detected at around *m*/*z* 700–900 and a few lipid ions were detected around *m*/*z* 1400–1600. A few more peaks were detected in a lower mass range below *m*/*z* 700. The lipid classes corresponding to these mass ranges are specified in the Supplementary Materials (Tables S3 and S4).

Furthermore, we noticed that the majority of the DMs detected at 16 weeks were also discriminative at other time points as shown in the Supplementary Materials (Tables S1 and S2).

### 4.2. Assignment of Differentially Regulated Lipids

**Figure 8:**
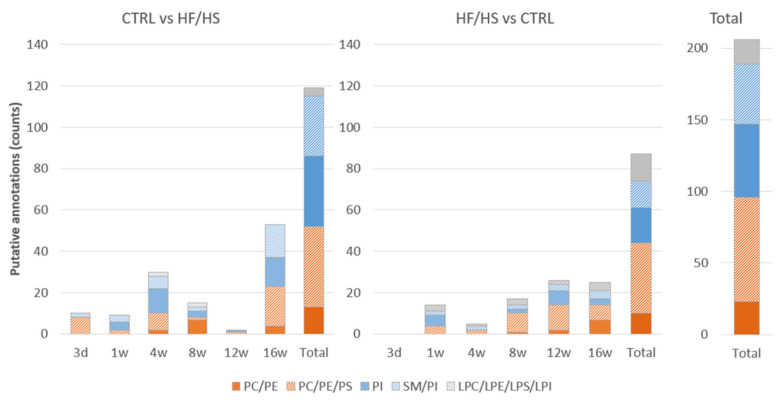
Lipid class categories of the putative annotations for the ROC-identified masses in the CTRL vs. HF/HS and HF/HS vs. CTRL comparisons at each time point and in total. Lipids could be putatively assigned either to phosphatidylcholines (PCs), phosphatidylethanolamines (PEs), and phosphatidylserines (PSs); or assigned to phosphatidylinositols (PIs) and/or sphingomyelins (SMs). A few lipid species were identified as LysoPC (LPC), LysoPE (LPE), LysoPS (LPS), and LysoPI (LPI).

A total of 206/549 ROC-identified DMs could be putatively annotated in both modes from all time points: 119 DMs in the CTRL vs. HF/HS comparison, and 87 DMs in the HF/HS vs. CTRL comparison. An overview of all ROC-detected DMs and their putative assignments can be found in Tables S3 and S4 as well as a summary of putative annotations in the Supplementary Materials (Table S5). The database with estimated assignments included a few possible annotations for the most detected masses. Therefore, the annotations are described in categories which include all possible assignments. As shown in Figure 8, the majority of the assigned DMs in both CTRL vs. HF/HS and HF/HS vs. CTRL comparisons were found to belong to phospholipids (putatively assigned to either phosphatidylcholines (PCs), phosphatidylethanolamines (PEs), phosphatidylserines (PSs), or to phosphatidylinositols (PIs), and/or sphingomyelins (SMs). Only a few DMs were identified as lysophospholipids (LPs, putatively assigned to either LysoPC (LPC), LysoPE (LPE), LysoPS (LPS), or LysoPI (LPI). 

The proportions of PCs, PEs and PSs were altered as early as 3 d in both directions of change, CTRL vs. HF/HS and HF/HS vs. CTRL, and at all time points. DMs specifically assigned to PEs or to the PC/PE category were detected at later time points especially from 8 w onwards in both directions. DMs identified as PIs were relatively more abundant in the CTRL vs. HF/HS comparison than the other direction (in total 34/119 (28.6%) vs. 17/87 (19.5%)). Alterations in lysophospholipids were relatively more abundant in the HF/HS vs. CTRL comparison in all time points starting from 1 w. 

## 5. Discussion

The aim of this study was to investigate whether an HF/HS diet can alter the lipidomic profile of the oviductal epithelium (OE) of mice and to study the patterns of these changes over time as the mice continue to consume the obesogenic diet and develop obesity. This was tested in an outbred Swiss mouse model, to increase the pathophysiological relevance to humans and livestock animals. 

The oviductal physiology is very difficult to study, especially in mice, due to the small size and therefore limited access to biological material such as the oviductal cells. For the first time, we were able to apply the use of MALDI mass spectrometry imaging technology on murine oviducts and examine the changes in the lipidomic profile of the OE due to feeding an HF/HS diet. Distinct differences could be detected already at 3 days and 1 week (w) of HF/HS diet feeding. These differences then remained relatively stable until 12 w before marked lipidomic changes could be detected again at 16 w. The majority of these differences were assigned to phospholipids, sphingomyelins as well as a few lysophospholipids. 

We only focused on the ampullar region of the oviduct, where fertilization and the first three days of early embryo development take place [69]. The ampulla has more secretory cells and is thus more active in de novo lipid synthesis compared to the isthmus [51,70]. Most of the lipid synthesis occurs in the epithelium [51]. In the present study, the detected masses exhibited very specific spatial distribution patterns which matched the corresponding histological layers of the oviduct, and indeed, a distinct lipid profile of the OE was noticeable in contrast with the surrounding stroma. Such a contrasting lipid profile of the OE could be due to the presence of secretory cells [60]. Distinct lipid profiles of epithelial cell layers compared to stroma have been reported in other tissues such as ovarian tissue [71] and prostate tissue [72]. 

Previous findings from our laboratory showed that mice are metabolically sensitive to high dietary saturated fat and high fructose consumption [25]. Blood analysis of the mice used in the present study was reported in our previous paper [41], showing a significant increase in blood total cholesterol concentrations already after feeding the HF/HS diet for 1 w. This acute increase in cholesterol levels was no longer detectable at 4 w of feeding, but the difference became distinct again at 8 w, 12 w and 16 weeks of feeding [41]. Similar duration-specific differences are also observed here in the present study. We saw a distinct lipidomic profile of the OE of the HF/HS-fed mice compared with the controls already at 3 days of feeding. This is shown by the increased average peak intensity of all detected signals in the spectra from the OE, and by the detection of DMs by the ROC analysis of the ion signals. The number of DMs gradually increased over time while the average peak intensity decreased in general, i.e., moving from a non-specific overall change in the OE lipid profile to a more specific change in certain lipid species. Furthermore, the results showed that the differences were stable from 1 w to 12 w of feeding. At 16 w of feeding, both the detected DMs and the average peak intensity were relatively high. This pattern of change also corresponds to the same distinct phases of systemic metabolic alterations reported in mice fed an HF/HS diet as described by Williams, Campbell [13]. In that study, an elevated glucose concentration and an acute phase response was reported already after 3 d of feeding. These effects were improved at 1 w of feeding and remained stable until 12 w. Inflammatory markers were undetectable during this period. At 16 w, Williams et al. reported another increase in glucose concentration and inflammation. They concluded that the development of diet-induced obesity and glucose intolerance consists of distinct phases, in which they proposed the time period between 1 w and 12 was an adaptation phase in the diet-induced metabolic changes. Adaptative mechanisms determining the difference in cellular fatty acid metabolism in short-term vs. long-term HF/HS diet feeding were also described by Ciapaite, van den Broek [73]. 

In addition, the pattern of changes in the overall average peak intensity of the OE can also be linked to the gene expression patterns of the OECs collected from the same mice, as previously published [41]. There, we showed that feeding an HF/HS diet significantly increased acute stress responses, as indicated by an increased level of oxidative stress and endoplasmic reticulum (ER) stress marker genes after only 3 d of feeding. This was followed by a phase (between 1 w and 8 w) in which these transcriptomic changes were temporarily normalized (became less distinct). But finally, a significant upregulation of genes associated with OS and ER stress as well as inflammation was detected again at 12 to 16 w in the HF/HS group [41]. The results of these studies show strong evidence of the sensitivity of the oviduct and OE to systemic metabolic changes at both transcriptomic and lipidomic levels. 

Further analysis showed that some of the detected discriminative masses (DMs) were common between the different feeding periods, and many of them were detected again at 16 w (see Tables S1 and S2). This can be seen as a validation of the results and strongly suggests that the detected DMs are specifically altered in response to the diet-induced metabolic alteration, potentially for a specific function or signalling response. Persistent alterations in common lipid species over different periods of HFD feeding were also reported in mice and rats in other tissues such as in the liver [74] and in the brain [75]. Moreover, the lipidomic changes occurring after chronic exposure (16 w) to the HFD were shown to be irreversible, as they persist even after switching to a chow diet [75]. Our findings show a relatively low number of common DMs between the early time points up until 12 w of feeding, which may indicate that the changes due to the HF/HS diet during this phase are variable and reversible. The low number of common DMs between the earlier time points may also be due to a high rate of turnover of lipids in the OE. The lipid profile in any tissue is a result of lipid uptake, metabolism and de novo synthesis. In vitro research in our laboratory using bovine oviductal epithelial cells cultured in a polarized set-up confirmed that OE cells are capable of de novo lipid synthesis [76]. 

The main organelle for lipid biosynthesis is the ER as it produces the majority of structural phospholipids [77]. As shown in our previous study, consuming the HF/HS diet leads to oxidative stress and ER stress in the OECs already after 3 d of feeding [41]. Although the cause–consequence relationship is not certain as the diet leads to a complex network of interacting changes, ER stress is known to be a key component of the pathogenesis of diet-induced lipotoxicity in many tissues and cells and diet-induced alterations in ER functions can indirectly alter the de novo lipid biosynthesis. Also, alterations in the blood lipid concentrations and degree of fatty acid saturation can directly influence the tissue lipid (selective) uptake and impact cellular lipid composition. In the present study, the detected DMs that were discriminative in the CTRL vs. HF/HS comparison (more abundant in the CTRL OE) were more abundant than those detected in the HF/HS vs. CTRL comparison (as confirmed in the Supplementary Materials (Figure S2). This may suggest that the de novo synthesis of many lipids is hampered in the OE of the HF/HS group, which applies to the detected DMs that were more abundant in the control OE compared to the HF/HS OE from as early as 3 days and up until 16 w. By contrast, DMs that were detected in the HF/HS vs. CTRL comparisons might be, at least in part, a direct effect of the type and concentration of the circulating lipids.

As a final step, we attempt to provide a general overview of the biological relevance and potential functional implications of the diet-induced lipidomic change in the OE. Identifying or annotating lipid *m*/*z* values in lipidomic analysis can be difficult due to structural ambiguities that may be present, such as bond type or the number of hydroxyl groups [78]. Species annotation may be based on assumptions in such cases, and a few putative annotations are often assigned to the same *m*/*z* value [78]. Hence, we focus here on the potential alteration in lipid classes and families, and their ratios, rather than on individual lipid classes. 

We found that phospholipids, namely PCs, PEs and PSs constitute almost 50% of the diet-induced lipidomic changes in the OE. This is true both in the CTRL vs. HF/HS and the HF/HS vs. CTRL comparison, i.e., some PCs, PEs, and PSs increase in abundance in the OEs due to diet, while others are reduced. Phospholipids are the major structural lipids in cell membranes [79] which are important for allowing e.g., fission and fusion, cell division, and intracellular membrane trafficking [80]. PCs represent 40–50% of the total cellular phospholipid content in all cells and play a vital role in cell functions and homeostasis [81]. For example, PC biosynthesis is required for the process of intracellular fatty acid internalization and incorporation into triacylglycerols in lipid droplets. This mechanism helps to reduce the impact of lipotoxicity [82]. Alteration in PC biosynthesis, as shown here in the OEs, may thus influence cell viability [83]. 

Several DMs in both directions of change were also assigned to PEs (see Tables S3–S5) or putatively assigned to the PC/PE or PC/PE/PS categories, especially at 8 w and afterwards (Figure 8). PE is the second most abundant phospholipid in mammalian membranes [81]. Alteration in PE biosynthesis can cause a shift in the PC/PE ratio which can have several crucial consequences. PEs and PCs regulate the expression of sterol regulatory-element-binding proteins (SREBPs) [84,85], which are binding proteins that regulate the de novo synthesis of lipids by cells [86]. PCs and PEs also form the two most abundant phospholipids of mitochondria, with PEs comprising about 40% of total phospholipids in the mitochondrial inner membrane [81]. It is known that mitochondrial dysfunction plays a role in metabolic disorders [87]. As mitochondrial lipids define the physical properties of mitochondrial membranes, any reduction in mitochondrial PEs, even if subtle, can alter mitochondrial functions [88] and has been linked with a profound alteration in mitochondrial morphology and embryonic lethality. Increased PC/PE ratio, due to decreased mitochondrial PE synthesis has even been shown to lead to severe impairment of cell survival and growth in mice [89]. Therefore, the differential abundance of PCs and PEs seen at the later time points in our study may be indicative of a shifted PC/PE ratio and may suggest an altered mitochondrial function and energy metabolism in the oviductal epithelium. 

Some DMs were (putatively) annotated as PSs. PS is a plasma membrane component and participates in many signalling pathways [90]. PS is highly enriched in the cytosolic leaflet of the membranes of recycling endosomes, which replenish the lipids and proteins of the plasma membrane, and it is essential for their function [91]. 

The second main category of lipids that were altered in the OE due to the HF/HS diet is that assigned to PI and SM. PIs were particularly abundant in the CTRL vs. HF/HS comparison starting from 1 w, indicating a reduced biosynthesis in the HF/HS group. PIs identify endocytic membranes and enable them to attract proteins from the cytosol, which are important for vesicle trafficking and cellular homeostasis [92], and thus play a key role in signalling and recognition [80]. Alterations in their expression may affect their role in endocytic pathways in the OE, with a potential impact on cellular homeostasis and cell-to-cell communication. The structural lipid SM, part of the sphingolipids class, was differentially regulated at all time points after the start of feeding the HF/HS diet [93]. SMs are inflammatory intracellular lipid species and, as for PCs, SMs have been reported to be crucial precursors of many biomolecules, such as prostaglandins and lipid mediators that play a role in many cell signalling pathways [94]. The backbone of SM is formed by CER, which is produced by the ER, through the action of SM synthases (SMSs) [93]. SM is involved in the regulation of endocytosis and receptor-mediated ligand uptake, ion channel and G-protein coupled receptor function, and protein sorting [95]. SM also has an important functional role as a precursor of sphingolipid signalling molecules [96]. Alterations in these lipids in OEs might thus have several functional consequences.

Lipids also play a key role as first and second messengers in signal transduction and molecular recognition processes [80]. Lysophospholipids, such as LPC and LPA, are a group of messenger lipids that are formed by the hydrolysis of phospholipids and sphingolipids [97]. In our study lysophospholipids LPC, LPE, LPI and LPS appeared to have a higher abundance in the HF/HS OE compared to the CTRL OE over the different time points, which may indicate that cell signalling processes might be affected due to the HF/HS diet. 

We did not focus on the putative annotations of the individual lipid species and their molecular weight and degree of saturation (Tables S3–S5) because of the potential ambiguity in these assignments. However, we have noticed that the identified DMs in the HF/HS vs. CTRL comparison are mostly higher in molecular weight than those in the CTRL vs. HF/HS direction of change. Belaz, Tata [98] have reported that the molecular weight (MW) of lipid ions in the bovine uterus and oviduct are linked to embryo receptivity and fertility. High-MW PCs (e.g., PC(38:7), PC(38:5), PC(40:7) and PC(40:6)) were linked with lower receptivity and low fertility, whereas the low-MW PCs and SMs (e.g., PC(32:1), PC(35:2), SM(34:1) and SM(34:2)) were associated with higher receptivity [98]. The potential role of these specific lipids and their MW in the oviduct under obesogenic conditions needs further investigation.

It is important to highlight that lipidomic alterations in the OEs can be reflected in the oviductal microenvironment and alter the interaction between the OE and the gametes and embryos. This may impact all the reproductive events that take place in the oviduct, such as fertilization and early embryo development. The lipid composition of the oviduct was shown to be reflected in the lipidomic profile of the oviductal secretions, including its extracellular vesicles content, which in turn has been shown to alter the lipid composition of the embryos upon direct contact [99]. Early embryos internalise and utilise lipids from their microenvironment [100], and thus the quantity and type of lipids determine embryo metabolic activity, ROS levels, developmental capacity, as well as embryonic transcriptomic profile and epigenetic programming [48,101,102,103]. The diet-induced alterations in the lipidomic profile of the OEs, detected as early as after 3 days of feeding, could therefore impact early embryo development and quality. Alterations in signalling molecules that are derived from lipids, such as prostaglandin synthesis in the oviduct, can also impact embryo development and quality [40,104], ultimately leading to lower pregnancy success or even to offspring health defects [105,106]. The magnitude of such impact is yet to be determined.

## 6. Conclusions

To the best of our knowledge, this is the first study describing the effect of an HF/HS diet on the lipid profile of the oviductal microenvironment in vivo. We can conclude that exposure to an obesogenic diet results in acute changes in the lipid profile in the OE after a short-term feeding period of only 3 days in mice, and thus even before the development of an obese phenotype. At the subsequent early time points, the effects appear to be variable and reversible but the changes in the lipid profile of the OE progressively increase and become more persistent after long-term exposure to the HF/HS diet. Functional annotation revealed a differential abundance of lipids, particularly phospholipids, sphingomyelins and lysophospholipids, which are involved in plasma membrane functions, cell signalling, mitochondrial functions and cellular homeostasis. These alterations appear to be not only due to the direct accumulation of the excess dietary fat in the OEs but also due to a reduction in the de novo synthesis of several lipid classes, most likely due to ER dysfunction.

## Figures and Tables

**Table 1:** Number of discriminative masses (DMs) at each time point for both acquisition modes and both comparisons. Both positive and negative acquisition modes were used to maximize the detection of different lipids.

Time Point	Negative Mode		Positive Mode		Total	Total	Total DMs
	HF/HS vs. CTRL	CTRL vs. HF/HS	HF/HS vs. CTRL	CTRL vs. HF/HS	HF/HS vs. CTRL	CTRL vs. HF/HS	
3 d	none	10	none	1	none	11	11
1 w	28	12	1	33	29	45	74
4 w	5	39	17	16	22	55	77
8 w	23	21	24	29	47	50	97
12 w	37	18	7	2	44	20	64
16 w	27	83	18	99	45	182	227

## Data Availability

The data presented in this study are available in the article and the Supplementary Materials.

## Supplementary Materials

The following supporting information can be downloaded at: https://www.mdpi.com/article/10.3390/biology12071016/s1, Figure S1. Representative ROC curve of *m*/*z* 553.34 detected in negative mode. Figure S2. Scatter plot showing the distribution of the average intensity of the peaks of each detected ion signal (mass) in time point 16 weeks.; Table S1: Common DMs detected at different time points in negative mode; Table S2: Common DMs detected at different time points in positive mode; Tables S3 and S4 in Appendix Excel file: List of putative annotation of the detected molecular species by MALDI MSI in HF/HS vs CTRL OE in Swiss mice in negative (Table S3) and positive (Table S4) acquisition mode; Table S5 in Appendix Excel file: Overview of putative annotations.
