# Supplementary material for: How the Oviduct Lipidomic Profile Changes over Time after the Start of an Obesogenic Diet in an Outbred Mouse Model
Source: Biology (Basel). 2023 Jul 17;12(7):1016. doi: 10.3390/biology12071016 (PMC10376370; doi:10.3390/biology12071016)
Supplement: Supplementary file 1 [file biology-12-01016-s001.zip › Supplementary files/Supplementary Figure S2.pdf]

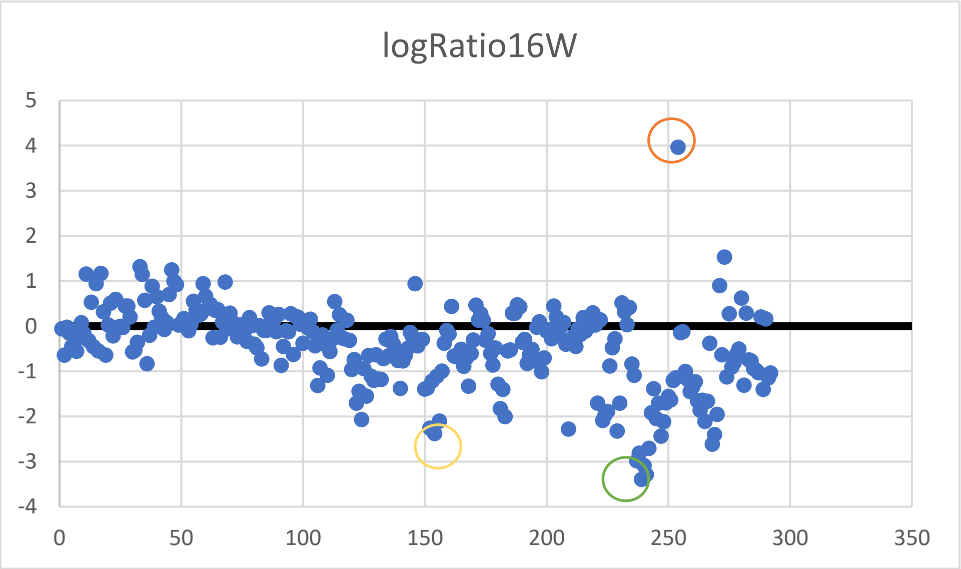

Supplementary Figure S2. Scatter plot showing the distribution of the average intensity of the peaks of each detected ion signal (mass) in time point 16 weeks. The ratio of the plot was calculated as HF/HS over CTRL. Therefore, the data points above the baseline are masses with a higher abundance in the HF/HS OE, whereas data points below the baseline are more abundant in the CTRL OE compared to the HF/HS OE. The green and yellow highlighted dots were detected as DMs in the ROC analysis in the CTRLvsHF/HS direction. The orange highlighted dot was identified as DM in the ROC analysis in the HF/HSvsCTRL direction.
