# Supplementary material for: How the Oviduct Lipidomic Profile Changes over Time after the Start of an Obesogenic Diet in an Outbred Mouse Model
Source: Biology (Basel). 2023 Jul 17;12(7):1016. doi: 10.3390/biology12071016 (PMC10376370; doi:10.3390/biology12071016)
Supplement: Supplementary file 1 [file biology-12-01016-s001.zip › Supplementary files/Supplementary Table S1.pdf]

Supplementary Table S1. Common DMs detected at different time points in negative mode.

Period		3d	1w	4w	8w	12w	16w
	**Total**	**10**	**40**	**44**	**43**	**55**	**110**
**3d**	**10**	10	0	1	2	2	6
**1w**	**40**		40	11	2	16	22
**4w**	**44**			44	14	13	35
**8w**	**43**				43	13	31
**12w**	**55**					55	36
**16w**	**110**						110
