# Supplementary material for: How the Oviduct Lipidomic Profile Changes over Time after the Start of an Obesogenic Diet in an Outbred Mouse Model
Source: Biology (Basel). 2023 Jul 17;12(7):1016. doi: 10.3390/biology12071016 (PMC10376370; doi:10.3390/biology12071016)
Supplement: Supplementary file 1 [file biology-12-01016-s001.zip › Supplementary files/Supplementary Table S2.pdf]

**Supplementary Table S2**. Common DMs detected at different time points in positive mode.

| **Period** | | **3d** | **1w** | **4w** | **8w** | **12w** | **16w** |
|---|---|---|---|---|---|---|---|
| | **Total** | **1** | **33** | **33** | **53** | **9** | **117** |
| **3d** | **1** | 1 | 1 | | 1 | | 1 |
| **1w** | **33** | | 33 | 2 | 8 | | 31 |
| **4w** | **33** | | | 33 | 8 | 1 | 21 |
| **8w** | **53** | | | | 53 | 1 | 25 |
| **12w** | **9** | | | | | 9 | 1 |
| **16w** | **117** | | | | | | 117 |
