# Supplementary figures and images for: How the Oviduct Lipidomic Profile Changes over Time after the Start of an Obesogenic Diet in an Outbred Mouse Model
Source: Biology (Basel). 2023 Jul 17;12(7):1016. doi: 10.3390/biology12071016 (PMC10376370; doi:10.3390/biology12071016)

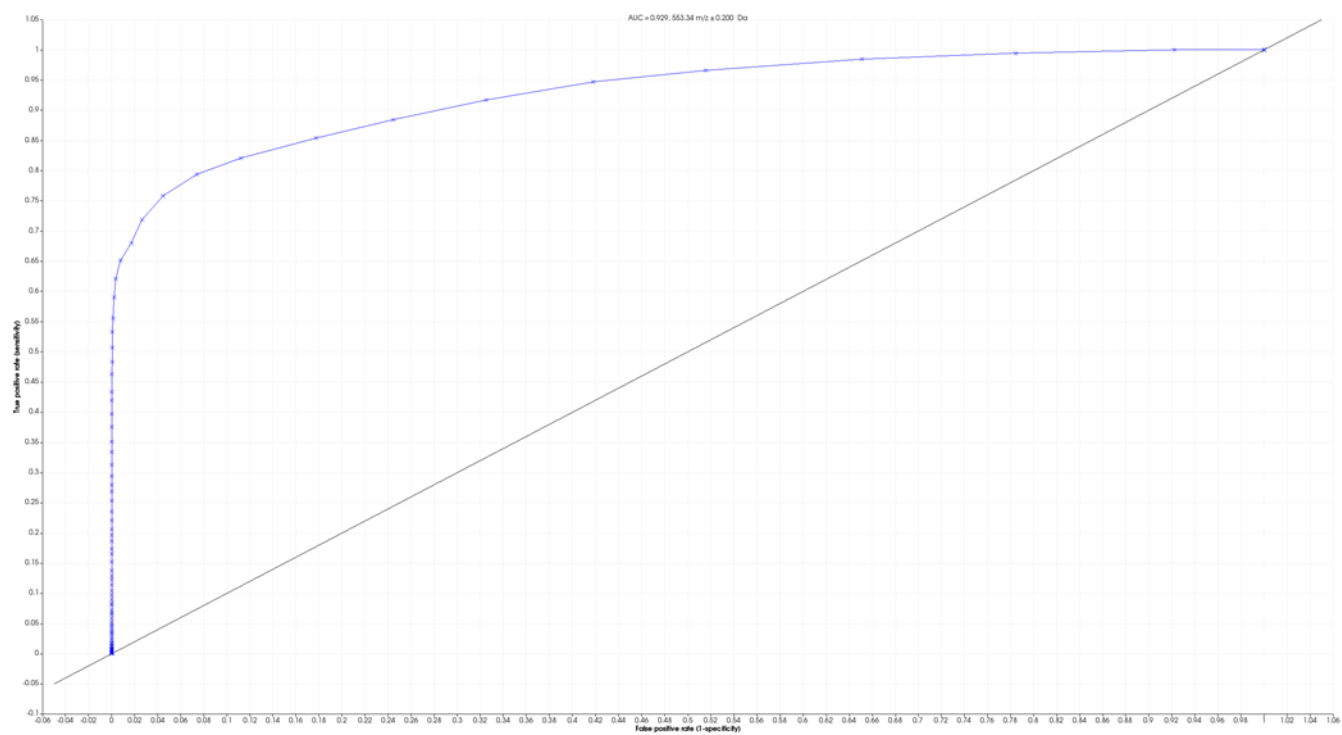

**Supplementary Figure S1.** Representative ROC curve of m/z 553,34 detected in negative mode.

Supplement: Supplementary file 1 [file biology-12-01016-s001.zip › Supplementary files/Supplementary Figure S1.pdf]
